# Antioxidant and Tyrosinase Inhibitory Activities of Seed Oils from* Torreya grandis* Fort. ex Lindl.

**DOI:** 10.1155/2018/5314320

**Published:** 2018-09-18

**Authors:** Hong-Xin Cui, Fang-Fang Duan, Shan-Shan Jia, Fang-Rong Cheng, Ke Yuan

**Affiliations:** ^1^College of Pharmacy, Henan University of Chinese Medicine, Zhengzhou 450046, China; ^2^Collaborative Innovation Center for Respiratory Disease Diagnosis and Treatment & Chinese Medicine Development of Henan Province, Zhengzhou 450046, China; ^3^Jiyang College of Zhejiang Agriculture and Forestry University, Zhu'ji 311800, China

## Abstract

*Torreya grandis* Fort. ex Lindl. is a plant belonging to the Taxaceae family and* Torreya grandis *cv. Merrillii is the only grafted and thoroughbred species belonging to this species. In this study, we extracted five different seed oils, including* T. grandis* seed oil (TGSO),* T. grandis* “Xiangyafei” seed oil (XYSO),* T. grandis* “Zhimafei” seed oil (ZMSO),* T. grandis *“Majus”seed oil (TGMSO), and* T. grandis* “cunguangfei” seed oil (CGSO) using physical pressure. The resulting extracts were analyzed to determine their fatty acid composition, antioxidant activity, and inhibitory activity towards tyrosinase. The results of the antioxidant activity assays revealed that XYSO and ZMSO exhibited much greater DPPH radical scavenging activity and ferric reducing power than TGSO. Notably, all five of the seed oils showed dose-dependent inhibitory activity towards tyrosinase. XYSO and TGSO gave the highest activities of all of the seed oils tested in the current study against monophenolase and diphenolase, with IC_50_ values of 227.0 and 817.5*μ*g/mL, respectively. The results of this study show that wild TGSOs exhibit strong antioxidant and tyrosinase inhibition activities. These results therefore suggest that wild TGSOs could be used as a potential source of natural antioxidant agents and tyrosinase inhibitors.

## 1. Introduction

Tyrosinase (EC 1.14.18.0) is a copper-containing mixed-function oxidase that is ubiquitously expressed in animals, plants, and microorganisms. Furthermore, tyrosinase is a key rate-limiting enzyme that can catalyze enzyme browning and melanin synthesis. Tyrosinase also exhibits monophenolase and diphenolase activities, which catalyze the hydroxylation of L-tyrosine to L-DOPA and the oxidation of L-DOPA to dopaquinone, which can undergo nonenzymatic polymerization to give dark pigments [1, 2]. In humans, the overexpression of tyrosinase leads to the overproduction of melanin in the skin, which can trigger hyperpigmentation effects such as freckles, melasma, age spots, and melanoma [3]. Free radicals also play an important role in the biosynthesis of melanin, and several studies have shown that free radicals are involved in the catalytic reactions of tyrosinase to give dopaquinone, which can undergo various oxidation reactions. Increasing in the number or the activity of the free radicals present in living systems can therefore lead to an increase in the production of melanin [4, 5]. It is noteworthy that antioxidants such as vitamin C and Trolox have been reported to exhibit potent inhibitory activities towards tyrosinase and melanin production and have consequently been used to inhibit melanogenesis [6]. Materials capable of inhibiting the activity of tyrosinase have attracted worldwide interest from researchers working in a variety of different fields, including biological medicine, agricultural sciences, chemistry, and pharmacology. Tyrosinase inhibitors capable of inhibiting the biosynthesis of melanin are used currently in various hyperpigmentation and cosmetic agents to control the formation of freckles [7, 8].

From an agricultural perspective, the overexpression of tyrosinase is related to the discoloration of some fruits and vegetables, as well as changes in their flavor and nutritional characteristics, which can have an adverse impact on their commercial value. The development of new biocompatible tyrosinase inhibitors capable of reducing the production of quinone and hydroquinone to prevent enzymatic browning and improve the color and nutritional characteristics of food is therefore highly desirable [9, 10]. Several synthetic tyrosinase inhibitors have been reported in the literature, such as kojic acid, which is widely used as a skin-whitening agent in cosmetics. However, the use of this compound in this context has been associated with several serious side effects, including erythema and contact dermatitis [11, 12]. Further research is therefore required to identify natural and biocompatible tyrosinase inhibitors that can be used as whitening agents and antibrowning agents in the cosmetic and food industries.

*Torreya grandis* Fort. ex Lindl. are plants belonging to the Taxaceae family, which are also known as “chiguo”. Six different species and two varieties of* Torreya* have been reported to date. Three of these species (*Torreya fargesii*,* Torreya fargesii *var. yunnanensis, and* Torreya grandis*) and two varieties (*Torreya jackii and Torreya grandis *var. Jiulongshanensis) can be found in China; one species (*Torreya nucifera*) can be found in Japan; and two species (*Torreya taxifolia *and* Torreya californica*) can be found in America [13].* Torreya grandis*, which has been cultivated in China for more than 1500 years, is a rare and unique species, which is mainly distributed across the hilly areas of subtropical China, which is located in Zhejiang, Fujian, Anhui, Jiangxi, Jiangsu, Hunan, Hubei, Guizhou, and other places in China; vast territories were distributed, among them,* Torreya grandis *Fort. ex Lindl is mainly distributed in Zhuji, Zhejiang province. In recent years, a large number of ancient trees of* Torreya grandis *Fort. ex Lindl have been found in this area; among them, there are 42 ancient trees over a thousand years old, and the oldest one has reached 1500 years [14, 15].* Torreya grandis* cv. Merrillii is the only grafted and thoroughbred species of* Torreya grandis *Fort. ex Lindl, which has a wide variety of seedling species because of its broad range of characteristics.* Torreya grandis *Fort. ex Lindl is well known for its edible seeds of* Torreya grandis,* which have a unique nutty flavor and high nutritional value, leading to their use in traditional Chinese medicine [14, 16, 17]. It is noteworthy that there are still four kinds of* Torreya grandis* of wild mutation variety of Zhimafei, Cunguangfei, Xiangyafei, and Dayuanfei, in China, and that the seeds of these wild species have comparable flavor and quality characteristics to those of cultivated* Torreya grandis*. However, the seeds of these wild plants are unsuitable for human consumption, and these species have consequently been abandoned without reasonable utilization. Previous studies have demonstrated that* Torreya grandis* seeds have multiple biological properties including antioxidative, anti-inflammatory, antiatherosclerosis, antiviral, antifungal, antitumor and antihelminthic activities, because of their rich nutritional content and numerous bioactive fatty acid, protein, vitamin, and mineral components [16–20]. With this in mind, we investigated the inhibitory activities of TGSO and four wild* Torreya grandis *seed oils (TGSOs) towards tyrosinase to provide a platform for the rational utilization of wild* Torreya grandis.*

## 2. Materials and Methods 

### 2.1. Chemicals

1,1-Diphenyl-2-picrylhydrazyl (DPPH), tripyridyltriazine (TPTZ), 6-hydroxy-2,5,7,8- tetramethylchroman-2-carboxylic, Trolox, and mushroom tyrosinase (3610 U/mg,* Agaricus bisporus*) were purchased from Sigma (St. Louis, MO, USA). Kojic acid, L-ascorbic acid, L-tyrosine, and L-DOPA (L-3,4-dihydroxyphenylalanine) were obtained from Aladdin Co., Ltd (Shanghai, China). All of the other chemicals and solvents used in this study were purchased as the analytical reagent grade from Huadong Medicine Co., Ltd (Zhejiang, China).

### 2.2. Collection and Preparation of Plant Material

Four kinds* T. grandis* of wild mutation variety of Zhimafei, Cunguangfei, Xiangyafei, and Dayuanfei and only one cultivar of* Torreya grandis* after grafting were collected from Zhuji, Zhejiang province, China, in October, 2015. These seeds were subsequently identified and authenticated by Professor Dr. Pinzhang Deng at the Forestry Bureau Zhuji, Zhejiang Province. After authentication, the seeds were cleaned, hulled, and immediately dried in a microwave oven (three space heats for 1 min), before being placed in a drying oven at 65°C for 6 h. Twenty-gram samples of the dried seeds were then placed in a QYZ-230 fully automatic hydraulic oil press (Shandong, China) and pressed to obtain the squeezed seed oils.

### 2.3. GC-MS Analyses of Squeezed Seed Oils

GC-MS analysis was conducted on a Bruker SCION SQ 456 Gas Chromatograph (Bruker Daltonics Inc., Billerica, MA, USA), which was attached to a mass spectrometer. The gas chromatograph system was equipped with a nonpolar, HP-5 capillary column (30 m × 250 *μ*m × 0.25 *μ*m), which was coated with 5% phenyl-95% dimethylpolysiloxane, as well as a flame ionization detector (FID). The oven temperature was programmed as follows: 45°C for 2 min; 45 to 270°C at a rate of 10°C/min; and 270°C for 6 min. Helium was used as the carrier gas at the rate 1 mL/min. All of the oil samples were dissolved in hexane at a concentration of 0.1 mg/*μ*L. The sample injection volume was set to 1 *μ*L with a split ratio of 1:10. L

Mass spectra were recorded in the EI mode at 70 eV with a scan quality range of 50-550 amu. Temperature of the ionization source is 200°C, voltage of the detector is 350 V. Mass spectra data for the different peaks were cross referenced with those found in the NIST27 and WILEY7 libraries to identify the different components. Each mass spectrogram corresponding to each chromatographic peak is qualitatively determined by computer chart-base; the relative content of each component is calculated by the peak area normalization method according to its total ion current chart.

### 2.4. In Vitro Antioxidant Activities

#### 2.4.1. DPPH Free Radical Scavenging Activity Assay

The DPPH free radical scavenging activities of the seed oils were measured using a procedure from the literature with minor modifications [21]. The samples were evaluated in 96-well microtiter plates. Stock solutions of the seed oils were prepared at a concentration of 1 mg/mL in dimethyl sulphoxide, DMSO. Standard solutions of Trolox were prepared for concentrations in the range of 0–2.5 mM. Two hundred microliter aliquots of each sample solution were separately added to the 96-well microtiter plate, followed by 50 *μ*L of a DPPH solution (1 mM in 50% ethanol), and the resulting mixtures were incubated in the dark for 45 min at room temperature. The absorbance of each well was then measured at 517 nm using an Infinite M 200 Microplate Reader (Tecan, Männedorf, Switzerland). The DPPH free radical scavenging potential was calculated using the following formula:

(1) DPPH  free  radical  scavenging activityI%=1−Ap−AcAmax×100%

where A_p_ is the absorbance of the sample, A_c_ is the absorbance of vehicle, and A_max_ is the absorbance of the DPPH solution. Each sample was tested three times (n=3). The concentration of DPPH was calculated using a Trolox standard curve and expressed as the Trolox equivalent antioxidant capacity (TEAC) with units of mmol Trolox equivalents/g.

#### 2.4.2. Ferric Reducing Power (FRAP) Assay

The FRAP assay was performed as described previously [22]. Fresh FRAP reagent was prepared each day by mixing 300 *μ*l of acetate buffer (pH 3.6, 0.3 mol/L) with 30 *μ*L of TPTZ (10 mmol/L) and 30 *μ*L of iron(III) chloride (20 mM). A small sample (10 *μ*L) was added to 300 *μ*L of the FRAP reagents described above, and the resulting mixture was incubated in the dark for 30 min. The absorbance of the mixture was then measured at 593 nm. Each sample was tested three times (n=3), and the results were expressed as TEAC with units of mmol Trolox equivalents/g.

#### 2.4.3. Total Reducing Power (TRP) Assay

TRP was determined using a previously published method [23]. Briefly, 500 *μ*L of each sample was treated with 2.5 mL of sodium phosphate buffer (0.2 mol/L, pH 6.6) and 2.5 mL of 1% potassium ferricyanide, and the resulting mixture was incubated at 50°C for 30 min. The sample was then treated with 2.5 mL of 10% trichloroacetic acid, and the resulting mixture was centrifuged at 4000* ×g* for 10 min. The upper layer (2.5 mL) was collected and mixed with 0.5 ml of 0.1% ferric chloride and 2.5 mL of distilled water. The absorbance of the resulting mixture was then measured at 700 nm against a blank. Several Trolox standards were prepared as calibration solutions with concentrations in the range of 0.04–0.80 mg/ml. Each sample was tested three times (n=3), and the results were expressed as TEAC with units of mmol Trolox equivalents/g.

### 2.5. Tyrosinase Inhibitory Activity Assay

#### 2.5.1. Monophenolase and Diphenolase Activity Assay

The tyrosinase inhibitory activity was evaluated using a previously reported method with minor modifications [24]. L-DOPA and L-tyrosine were used as substrates in this assay. Forty microliters of L-DOPA (10 mM, for the diphenolase activity assay) or L-tyrosine (5.0 mM, for the monophenolase activity assay) was mixed with 80 *μ*L of phosphate buffer (0.1 M, pH 6.8) in a 96-well microtiter plate, and the resulting mixture was incubated for 10 min at 37°C. Forty microliters of seed oil (50, 100, 200, 400, and 800 *μ*g/mL in 50% DMSO) and 40 *μ*L of mushroom tyrosinase (250 U/mL, in PBS) were then added to each well on the plate, and the absorbance characteristics of the resulting mixtures were measured at 475 nm (for the diphenolase activity) or 490 nm (for the monophenolase activity) using an ELISA reader (Infinite M 200, Swiss Tecan) at 60-second intervals over a period of 120 min. PBS was added instead of the test sample as a blank control and kojic acid (50 *μ*g/mL) and ascorbic acid (50 *μ*g/mL) were used as positive controls. The inhibition for each enzyme assay was calculated as follows:

(2) $$\begin{array}{c} \%\;\;Inhibition=\frac{\left({Abs}_{control}-{Abs}_{sample}\right)}{{Abs}_{control}}\times 100 \end{array}$$

Each experiment was carried out in triplicate (n=3). The IC_50_ value, i.e., the concentration of seed oil, kojic acid, or ascorbic acid required to inhibit the activity of the enzyme by 50%, was calculated from the dose-response curves by nonlinear regression analysis using version 5.0 of the GraphPad Prism software version 5.0 (GraphPad software Inc., San Diego, CA, USA).

#### 2.5.2. Kinetic Analysis of the Inhibition of Tyrosinase

The inhibition kinetics of the different seed oils (50, 100, 200, 400, and 800 *μ*g/mL) towards tyrosinase were determined using different concentrations (0, 0.5, 1, 1.5, and 2 mM) of L-DOPA and L-tyrosine. The type of inhibition was analyzed using a Lineweaver–Burk plot. All of the experiments were performed in triplicate. The enzyme-inhibitor dissociation constant (K_i_) and K_m_ values were obtained from the slope of each plot and the y-intercept versus the seed oil concentration, respectively [25].

### 2.6. Statistical Analyses

Data were expressed as the mean values ± SD. Statistical analyses were performed using SPSS 19.0 (SPSS Inc., Chicago, IL, USA) and version 5.0 of the GraphPad Prism Software (GraphPad).

## 3. Results and Discussion

### 3.1. GC-MS Results of the Five Squeezed Seed Oils

**Figure 1:**
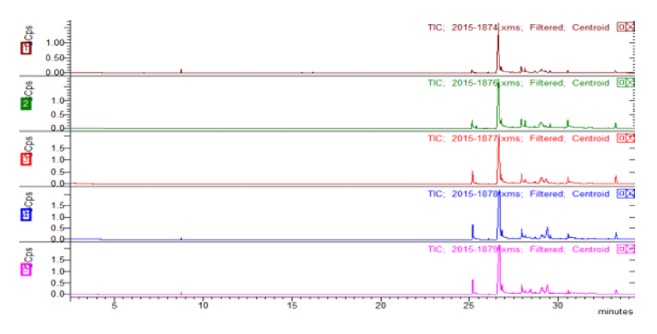
Total ion chromatograms of the seed oils obtained from the five different varieties of* T. grandis*. 1: TGSO, 2: XYSO, 3: ZMSO, 4: TGMSO, and 5: CGSO.

Plant seed oils continue to receive growing interest from researchers working in a variety of different fields, including chemists, pharmacists, and physicians because of their potential applications in the pharmaceutical, cosmetics, and food industries [26].* Torreya grandis* cv. Merrillii is the only grafted and thoroughbred species of* Torreya grandis *Fort. ex Lindl to have been reported in the literature to date and its seeds possess a unique flavor, as well as being rich in nutrients. Despite their huge potential, wild* Torreya grandis* are currently poorly utilized, especially those that cannot be eaten, with most of these plant resources simply going to waste. To develop a better understanding of the composition of the seed oils derived from these plants, we conducted a series of gas chromatography experiments, and the results are shown in Figure 1 and Table 1. The results showed that the chemical compositions of the seed oils derived from the five different varieties of* Torreya grandis* were roughly the same, although there were some subtle differences. Oleic acid, linolenic acid, and palmitic acid are the main fatty acids compositions in the five seed oils, in particular, the content of conjugate linoleic acid is relatively the highest, and it has been reported that linolenic acid has obvious tyrosinase inhibition activity [27]. The nonedible wild* Torreya grandis* seeds, such as XYSO and ZMSO, showed similar chemical compositions to TGSO. Although it would not be possible to directly commercialize the seed oils obtained from wild* Torreya grandis*, they could potentially be used to develop edible oils.

### 3.2. Antioxidant Results of the Five Squeezed Seed Oils

Oxidation is essential to many living organisms for the production of energy to fuel biological processes. However, free radicals and several other reactive oxygen species are continuously produced* in vivo* as a consequence of oxidation processes, and these reactive species can result in tissue damage and cell death. The oxidative damage caused by free radicals plays a major contributory role in aging and numerous debilitating diseases, including diabetes, cirrhosis, and cancer [28]. Although several enzymes (e.g., superoxide dismutase) and antioxidant compounds (e.g., ascorbic acid) can protect most organisms against free radical-induced oxidative damage, these systems cannot prevent this damage completely. The use of antioxidant supplements or the consumption of foods containing antioxidants can therefore be used as a strategy to reduce the level of oxidative damage [29]. The antioxidant activities of the seed oils obtained from the five different varieties of* Torreya grandis* evaluated in the current study were measured using DPPH, FRAP, and TRP assays. The DPPH assay, which measures the scavenging ability of an antioxidant towards the stable DPPH free radical, is one of the most commonly used methods for evaluating the radical scavenging activity of an antioxidant. Furthermore, it is well known that radical scavenging ability correlates well with antioxidant activity [30]. Ferric reducing ability (FRAP) and the TRP are commonly used methods for assessing the reducing power of an antioxidant in vitro. The research groups of Yen and Siddhuraju reported that there is a close relationship between the reducing power and antioxidant activity of antioxidants, and that the antioxidant capacity generally increases with increasing reducing power [31, 32]. In this way, it is therefore possible to evaluate the antioxidant ability of an antioxidant based on its reducing power. As shown in Table 2, the antioxidant capacities of the seed oils obtained from the five different varieties of* Torreya* were weaker than that of the positive control ascorbic acid. However, the DPPH scavenging capacities of the seed oils obtained from the five different varieties of* Torreya* were similar. The FRAP and TRP properties of the different seed oils also showed a similar trend, and there were significant differences (P<0.05) between the seed oils obtained from the different varieties of* Torreya* in one assay. The DPPH free radical scavenging abilities of the oils tested in the current study were found to be of the following order: ascorbic acid > XYSO > TGSO > ZMSO > TGMSO > CGSO; whereas the FRAP and TRP properties of the five different squeezed oils were of the order: ascorbic acid > TGSO > XYSO > ZMSO > TGMSO > CGSO.

### 3.3. Tyrosinase Inhibitory Activities of the Five Squeezed Seed Oils

**Figure 2:**
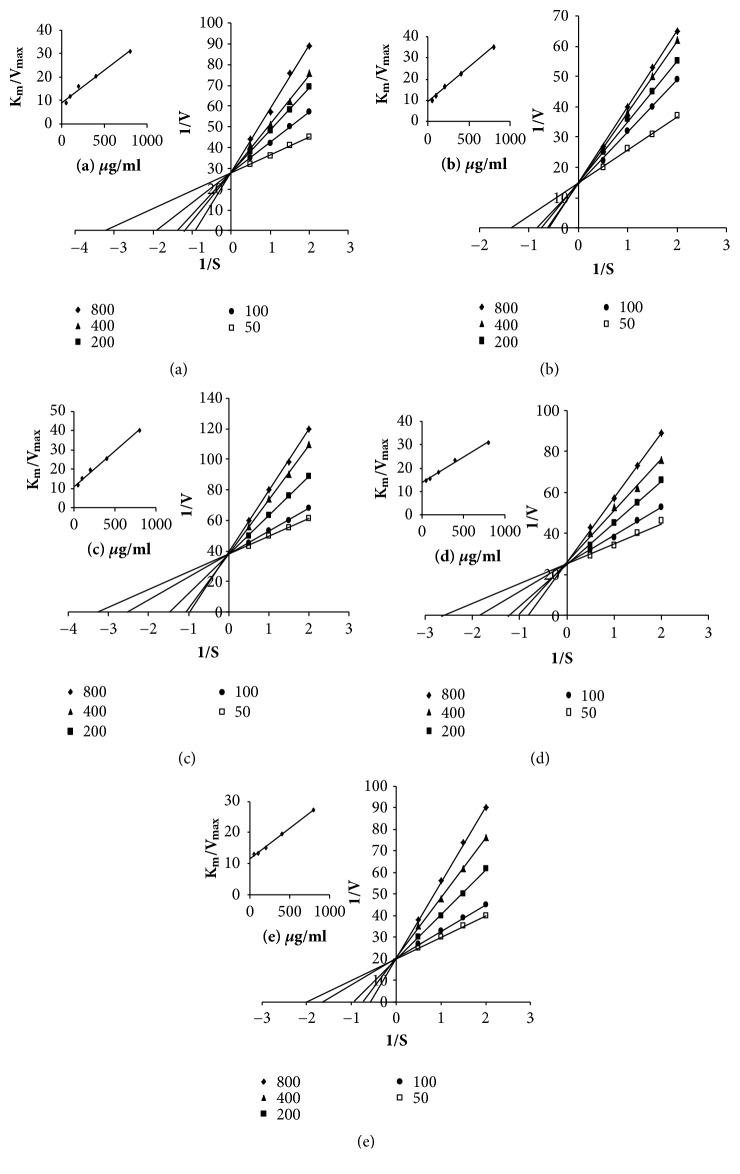
Determination of the mode of tyrosinase inhibition for the seed oils derived from the five different species of T. grandis. Lineweaver–Burk plots for the tyrosinase activity (diphenolase activity) using L-DOPA as a substrate with seed oil concentrations of 50 (□), 100 (●), 200 (■), 400 (▲), and 800(◆) *μ*g/ml. (The inset represents the slope (Km/Vmax) versus seed oil concentration (*μ*g/ml).) (a) TGSO, (b) XYSO, (c) ZMSO, (d) TGMSO, and (e) CGSO.

The effects of the five different varieties of seed oils on the oxidation reactions of L-DOPA and L-tyrosine using mushroom tyrosinase were determined experimentally. Tyrosinase is an important enzyme that plays a big role in melanogenesis within melanocytes. Furthermore, melanogenesis plays an important role in protecting the skin from sun-related injuries and is principally responsible for skin color. However, abnormal hyperpigmentation problems such as freckles, chloasma, and lentigines can have an adverse impact on the physical appearance of skin, representing serious esthetic problems [28]. Tyrosinase activity is one of the major targets used to screen for new inhibitors of melanin biosynthesis. Tyrosinase inhibitors can be used to achieve healthy skin-whitening and have consequently been widely used in cosmetics, medicine, food, and agriculture, where there is a growing interest in the development of safe and effective tyrosinase inhibitors from natural plants [33]. Kojic acid is a metabolite of fungal that exhibits inhibitory activity towards tyrosinase. The inhibitory effects of kojic acid in this context have been attributed to its ability to chelate copper at the active site of this enzyme. For this reason, kojic acid is often used as a positive control to discover new tyrosinase inhibitors, as well as being used as a cosmetic skin-whitening agent and a food additive for preventing enzymatic browning. But, its uses are restricted because of its several side effects [34]. The effects of the five different varieties of squeezed oil on the diphenolase activity of mushroom tyrosinase were assayed using L-DOPA as a substrate with kojic acid as a reference compound. The results revealed that all five of the oils inhibited the oxidation of L-DOPA in a dose-dependent manner (Figure 2). However, the inhibitory effects of the oils were less than that of kojic acid. Among the five oils, TGSO, XYSO, and ZMSO showed the greatest activities at highest concentration (800*μ*g/mL). However, at the lowest concentration (50 *μ*g/mL), DYSO and CGSO exhibited the best activity. The IC_50_ values of the five different oils against tyrosinase were less than 400*μ*g/mL.

**Figure 3:**
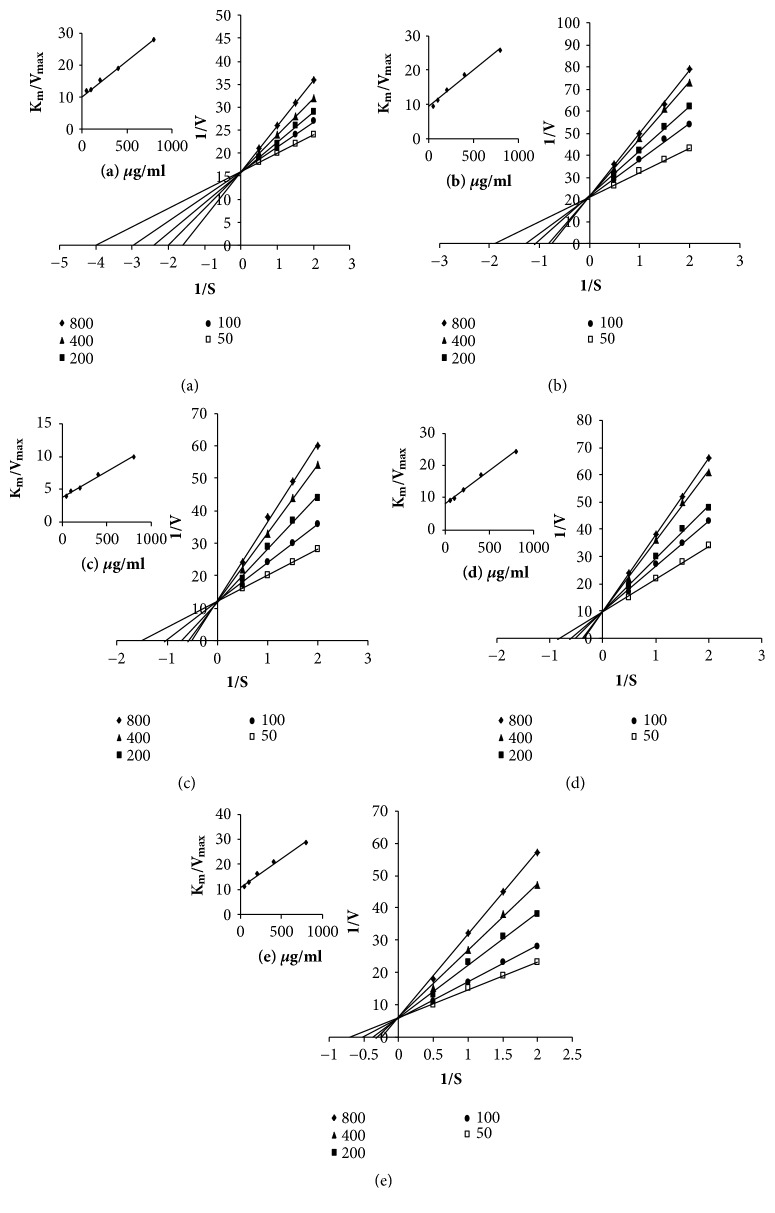
Determination of the mode of tyrosinase inhibition for the seed oils derived from the five different species of T. grandis. Lineweaver–Burk plots for the tyrosinase activity (monophenolase activity) using L-tyrosine as a substrate with seed oil concentrations of 50 (□), 100 (●), 200 (■), 400 (▲), and 800 (◆) *μ*g/ml (The inset represents the slope (Km/Vmax) versus seed oil concentration (*μ*g/ ml).) (a) TGSO, (b) XYSO, (c) ZMSO, (d) TGMSO, and (e) CGSO.

Ascorbic acid is commonly used as a tyrosinase inhibitor because of its antibrowning effect, and this compound has also been used as positive reference compound to evaluate new monophenolase inhibitors [35]. Ascorbic acid can also be used to affect the chemical reduction of dopaquinone, thereby avoiding the formation of dopachrome and melanin by the subsequent reduction of o-dopaquinone to L-DOPA. However, the application of ascorbic acid in this context has been limited by its poor stability [36]. The effects of the five different seed oils on monophenolase activity were studied using L-tyrosine as a substrate and ascorbic acid as a positive control. As shown in Figure 3, all five of the seed oils tested in the current study exhibited concentration-dependent inhibitory activity towards the tyrosinase-mediated oxidation of L-tyrosine. However, the inhibitory activities of all five oils were less than that of ascorbic acid, especially at a high concentration (800 *μ*g/mL). The IC_50_ values of the seed oils derived from the five different varieties of* Torreya* were greater than 800*μ*g/mL. Lastly, as is known in Figure 2, the five different seed oils showed slightly better diphenolase activity than their monophenolase activity.

The seed oils from the five different varieties of* Torreya grandis* all exhibited potent inhibitory activity towards tyrosinase (especially TGSO and XYSO), most likely because of their strong antioxidant activity. It is possible that the free radical scavenging activities of these seed oils prevented the initiation of a radical chain, thereby inhibiting the activity of tyrosinase by reducing the supply of oxygen during the tyrosinase reaction. These could be antioxidant active components present in* Torreya* seed oils, such as conjugated linoleic acid and Oleic acid, which could reduce the catalytic activity of tyrosinase by preventing the active effect of oxygen on tyrosinase.

### 3.4. Inhibition Kinetics of the Five Squeezed Seed Oils

The inhibition kinetics of the five different oils were analyzed using the Lineweaver–Burk method to determine their inhibitory activities towards diphenolase and monophenolase, and the corresponding plots are shown in Figures 2 and 3. The results revealed that the K_m_ values of the oils varied considerably with increasing concentration, whereas their V_max_ values remained largely unchanged. This result indicated that all five oils were behaving as competitive inhibitors of diphenolase and monophenolase. The inhibition constants (K_i_) of the different oils are shown in Table 3, together with their K_m_ and V_max_ values.

## 4. Conclusions

It is generally believed that there are four main methods for inhibiting tyrosinase activity: (1) affecting the chelating activity of copper at the active site of the enzyme, thereby preventing the binding of copper ions to oxygen, leading to the irreversible deactivation of tyrosinase [37, 38]; (2) the introduction of a powerful antioxidant, thereby preventing the activation of oxygen by tyrosinase [39, 40]; (3) the use of a free radical scavenging agent to prevent the formation of melanins [41]; and (4) the use of competitive inhibitors. Compounds with high antioxidant and radical scavenging activities could inhibit tyrosinase [42]. The seed oils derived from the five different varieties of* Torreya grandis* in the current study all exhibited potent inhibitory activity towards tyrosinase (especially TGSO and XYSO), most likely because of their strong antioxidant activity. It is possible that the free radical scavenging activities of these oils prevented the initiation of a radical chain, thereby inhibiting the activity of tyrosinase by reducing the supply of oxygen during the tyrosinase reaction. There could be several antioxidants present in* Torreya* seed oils, which could reduce the catalytic activity of tyrosinase by preventing the active effect of oxygen on tyrosinase. However, only in vitro experiments showed that seed oil of the five T.* grandis* has the potential application as skin-whitening, the research has yet to be validated by animal experiments in vivo. Although the diphenolase IC_50_ are not particularly low in seed oil, it has no powerful inhibition of tyrosinase, but some of their constituents might be more effective and have strong whitening effect. Further in depth studies would be required involving the individual components to accurately determine which compounds are responsible for inhibiting this enzyme. These findings suggest that wild TGSOs (especially XYSO) exhibit significant antioxidant and inhibitory activities towards tyrosinase (especially diphenolase), and that they could therefore be used to develop skin-whitening and/or anti-food-browning agents. Notably, the yields of the wild TGSOs (XYSO and ZMSO were particularly outstanding) were similar to that of TGSO, highlighting the potential for wild seeds to be converted into edible oils.

## Figures and Tables

**Table 1:** Main chemical components of the seed oils obtained from the five different varieties of *T. grandis*.

**Compound**	**TGSO**	**XYSO**	**ZMSO**	**TGMSO**	**CGSO**
oleic acid	30.52%	19.58%	8.07%	12.45%	20.51%
linoleic acid	25.94%	21.67%	7.52%	15.49%	0
palmitic acid	3.23%	8.65%	7.60%	0	7.27%
octadecanal	1.27%	10.77%	9.95%	33.50%	0
linolenic acid(LNA)	5.35%	4.21%	5.79%	0	2.02%
Cis-8,11,14-Eicosatrienoic acid	2.67%	3.24%	3.01%	1.13%	0.86%
Cis-5,11,14-Eicosatrienoic acid	13.93%	11.56%	10.33%	3.93%	3.57%
Oil yield	59.47%	56.99%	51.67%	35.01%	29.89%

**Table 2:** Result of the antioxidant activity assays.

**Assays**	**TGSO**	**XYSO**	**ZMSO**	**TGMSO**	**CGSO**	**Ascorbic acid**	**Kojic acid**
DPPH(mgTEs/g oil)	42.12±4.36^c^	56.73±0.67^b^	35.35±0.99^d^	24.87±0.88^e^	22.34±0.71^f^	194.56±3.22^a^	-
FRAP(mgTEs/g oil)	690.32.±41.32^b^	667.89±50.21^c^	588.91±33.21^d^	401.67±46.04^e^	355.56±44.87^f^	1135.41±55.68^a^	-
TRP(mgTEs/g oil)	199.76±35.12^b^	186.77±31.23^c^	151.03±30.76^d^	111.43±11.56^e^	109.61±23.98^f^	385.98±41.21^a^	-

TEAC values (mg TEs/g) of the antioxidants derived from the five different varieties of* T. grandis*.

**Table 3:** The test results of tyrosinase inhibition activity and kinetic parameters.

**Name**	**V**_**max**_ **(**△**OD**_**475**_**/min)**	**IC**_**50 **_ **(** ***μ*** **g/ml)**	**K**_**m**_ **(mM)**	**K**_**i**_ ** (** ***μ*** **g/ml)**
Diphenolase				
TGSO	0.167	237.42±2.23	1.92	637.79±6.31
XYSO	0.147	227.01±2.68	1.61	587.6±3.87
ZMSO	0.1	277.98±1.38	0.89	297.39±5.61
TGMSO	0.063	244.52±4.15	0.87	317.57±3.48
CGSO	0.083	326.63±2.55	0.77	280.74±4.26
Kojic acid	-	32.65±0.42	-	-
Monophenolase				
TGSO	0.066	849.42±4.37	0.72	481.87±6.84
XYSO	0.05	817.53±3.79	0.44	473.7±4.32
ZMSO	0.04	879.65±5.13	0.37	396.7±5.11
TGMSO	0.036	936.44±6.38	0.14	563.17±3.27
CGSO	0.027	968.28±6.52	0.22	443.43±4.44
Ascorbic acid	-	35.97±0.13	-	-

Summary of the inhibition kinetics of the antioxidants derived from the five different varieties of* T. grandis*. *Notes*. Data represent the mean values ± SD (n=3).

## Data Availability

The data used to support the findings of this study are available from the corresponding author upon request.

## Abbreviations

TGSO: * T. grandis* seed oil
XYSO: * T. grandis* “Xiangyafei” seed oil
ZMSO: * T. grandis* “Zhimafei” seed oil
TGMSO: * T. grandis* “Majus” seed oil
CGSO: * T. grandis* “cunguangfei” seed oil
DPPH: 1,1-Diphenyl-2-picrylhydrazyl
FRAP: Ferric reducing power
TPTZ: Tripyridyltriazine
Trolox: 6-Hydroxy-2,5,7,8-tetramethylchroman-2-carboxylic
DMSO: Dimethyl sulphoxide
TEAC: Trolox equivalent antioxidant capacity
PBS: Phosphate buffer
IC_50_: 50% inhibition concentration
K_i_: Enzyme-inhibitor dissociation constant
K_m_: Michaelis-Menten constant.
